# Recent Update on PCSK9 and Platelet Activation Experimental Research Methods: In Vitro and In Vivo Studies

**DOI:** 10.3390/jcdd9080258

**Published:** 2022-08-10

**Authors:** Meidi Utami Puteri, Nuriza Ulul Azmi, Salbiah Ridwan, Muhammad Iqbal, Tresni Fatimah, Tri Diana Puspita Rini, Mitsuyasu Kato, Fadlina Chany Saputri

**Affiliations:** 1 Department of Pharmacology-Toxicology, Faculty of Pharmacy, Universitas Indonesia, Depok 16424, Indonesia; 2 Humanics Program, School of Integrative and Global Majors, University of Tsukuba, Tsukuba 305-8575, Japan; 3 Department of Experimental Pathology, Faculty of Medicine, University of Tsukuba, Tsukuba 305-8575, Japan; 4 Magister Program, Faculty of Pharmacy, Universitas Indonesia, Depok 16424, Indonesia

**Keywords:** atherosclerosis, animal models, cardiovascular disease, experimental methods, myocardial infarction, platelet activation, proprotein convertase subtilisin/kexin type 9

## Abstract

Proprotein convertase subtilisin/kexin type 9 (PCSK9) is a crucial factor in the development and progression of cardiovascular diseases. PCSK9 has been demonstrated to modify LDL plasma levels and increase platelet activation, which promotes atherosclerosis, a defining feature of nearly all cardiovascular diseases. Platelet activation has been shown to promote and maintain the response to atherosclerosis development, from beginning to progression and exacerbation, which can lead to advanced cardiovascular events including myocardial infarction (MI) or death. Research on PCSK9 and platelet activation is currently underway with the main goal of reducing the risk of advanced cardiovascular events by preventing or slowing down atherosclerosis progression. Both in vitro and in vivo studies have been used to explore PCSK9 functions to develop new drugs targeting PCSK9. Finding the most suitable study models that represent the pathological and physiological systems found in humans is very important to achieving the goal. This review aimed to present a current and comprehensive overview of the experimental models that have been used to investigate the role of PCSK9 in platelet activation-induced atherosclerotic cardiovascular diseases.

## 1. Introduction

Proprotein convertase subtilisin/kexin type 9 (PCSK9) or firstly named as neural apoptosis-regulated convertase 1 (NARC-1) is a subtilisin-associated serine endo-protease that belongs to the proteinase K subfamily [1]. It is abundantly found in the liver, intestines, and kidneys and is involved in controlling low-density lipoprotein receptor (LDLR) expression and low-density lipoprotein-circulating cholesterol levels (LDL-c) [1]. PCSK9 regulates cholesterol metabolism by binding to hepatic LDLR and facilitating LDLR lysosomal degradation [2]. It may also influence cholesterol and triglyceride regulation in the intestine and adipose tissue [3], thus making it a potential candidate for drug therapy in cardiovascular diseases [2]. A number of research on cardiovascular-related diseases such as atherosclerosis, myocardial infarction (MI), heart failure, and angina have been conducted over the years [4]. The use of relevant research models that range from cell-based culture to animal models is required to mimic cardiovascular pathogenesis that results in valuable insight for novel therapeutic approaches [5]. Improvements for medical therapy are obtained from established studies that successfully provide new information on the causes of cardiovascular disease, one of which is the PCSK9 study on platelet activation. Effective studies of cardiovascular diseases related to PCSK9 require the use of appropriate cells and animal models. Therefore, we describe some of the in vitro and in vivo study models that have been employed in current PCSK9 and platelet activation research, as well as a possible in vivo study model that can be established for future PCSK9 and platelet activation research in this review.

## 2. Atherosclerosis, Platelet Activation, Myocardial Infarction (MI), and PCSK9

Atherosclerosis is a condition in which arteries constrict and stiffen due to plaque formation within the artery walls, which is caused by the buildup of lipid molecules, primarily low-density lipoprotein (LDL) molecules [6]. It has been studied that atherosclerosis is commonly initiated by a hypercholesterolemia condition [7]. When cholesterol-rich LDLs accumulate in the intima and activate the endothelium, the atherosclerotic process begins, which is then followed by monocyte and T cell recruitment that is facilitated by leukocyte adhesion molecules and chemokines [7,8]. As monocytes mature into macrophages, receptors for pattern recognition such as toll-like and scavenger receptors are amplified [6]. Internalization of lipoproteins is mediated by scavenger receptors, resulting in the formation of foam cells [6]. Toll-like receptors send out activating signals, which trigger the release of proteases, vasoactive chemicals, and cytokines [7,8]. Further, in response to environmental stimuli, T cells in lesion sites also produce T helper 1, which releases pro-inflammatory cytokines that cause local inflammation and promote plaque formation [6,7,8].

Next, platelets promote plaque formation through interacting with endothelial cells, circulating leukocytes (monocytes, neutrophils, dendritic cells, T cells), and progenitor cells [6,8]. Platelets support inflammatory cells in their route to the lesion site, where they release a cascade of inflammatory mediators that enhance and enrich the inflammatory state in atherosclerosis [6,8]. The increased inflammatory activity causes local proteolysis, plaque puncture, and thrombus generation, which all can contribute to ischemia and infarction [6,8]. Atherosclerosis is the cause of many advanced cardiovascular incidents and is the hallmark of practically all cardiovascular diseases including stroke and myocardial infarction (MI). MI is one of the world’s most common causes of death [9]. It belongs to the category of coronary artery disease, which includes 30–50% of patients with cardiovascular disease [4,9]. A reduction or complete cessation of blood flow to a region of the myocardium causes MI, often known as a “heart attack” [4]. It is commonly marked by a buildup of atherosclerosis in the artery walls, which might lead to plaque rupture and thrombosis [6]. These events could result in an ischemic state and myocardial necrosis [6].

PCSK9’s role in lipid homeostasis, particularly plasma LDL-c levels, was initially established in 2003 by Abifadel et al., who revealed that certain PCSK9 gene mutations can cause autosomal dominant hypercholesterolemia [10]. The importance of PCSK9 for LDL-c homeostasis was further highlighted when it was discovered that PCSK9 loss- and gain-of-function mutations cause hypo- or hypercholesterolemia in patients with devastating risks for atherosclerotic cardiovascular disease and its progression [11,12]. Furthermore, those who had PCSK9 dysfunctional mutations also had reduced LDL-c levels and an 88% decreased risk of coronary artery disease [13]. Despite very low LDL-c blood levels, a family history of PCSK9 dysfunctional mutations does not correspond to negative health outcomes [14]. Therefore, PCSK9 has been identified as a possible target for the development of innovative cardiovascular disease therapeutics.

The mechanisms whereby PCSK9 modulates LDL-c plasma levels have been previously identified. It all starts with circulating PCSK9 binding to the LDLR on the hepatocellular surface, which leads to LDLR breakdown in the lysosome [15,16]. As a result of the decreased level of LDLR on the hepatocellular surface, LDL uptake is reduced and LDL-c plasma levels rise [17,18]. On the other hand, the internalized LDLRs that are not coupled to PCSK9 are released back to the hepatocellular surface, increasing the amount of LDLRs and improving their ability to remove LDL particles from circulation [13]. PCSK9 has been linked to its possible role in preventing or reducing the progression of atherosclerotic cardiovascular illnesses due to its capacity to lower LDL-c plasma levels [2]. Furthermore, investigations have revealed that PCSK9 promotes inflammation and platelet activation, in addition to its role in lipid metabolism, which contributes to atherosclerosis [19,20].

**Figure 1:**
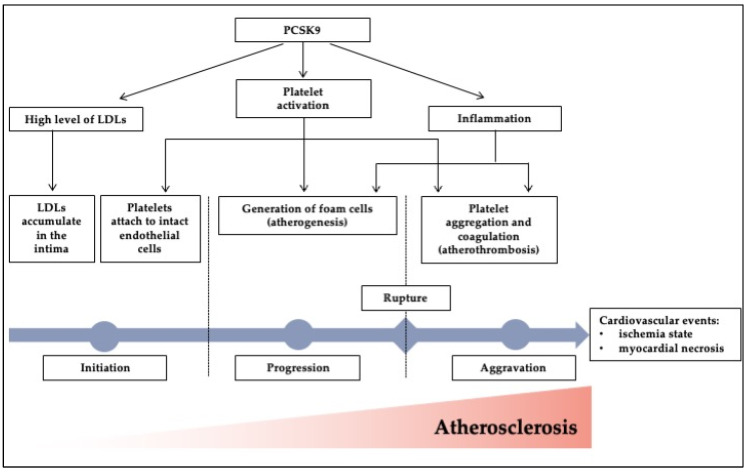
The roles of PCSK9 during atherosclerosis development. PCSK9 promotes an increase in LDL levels, platelet activation, and inflammation, all of which contribute to the progression of atherosclerosis. From initiation, progression, and aggravation, all of the processes work together to accelerate and sustain the progression of cardiovascular disease, which can lead to ischemia and, eventually, an MI event.

PCSK9 has been recently shown to bind directly to platelet CD36, triggering coagulation signaling cascades and enhancing ROS expression during atherosclerosis development [21]. CD36 is a glycoprotein membrane that is expressed by platelets, endothelial cells, adipocytes, myocytes, mononuclear phagocytes, hepatocytes, and some epithelia [22]. Moreover, CD36 is a receptor for thrombospondin 1 and similar proteins [22]. PCSK9 attaches to CD36, activating cPLA2 and platelet coagulation signaling pathways through the p38MAPK pathway, promoting thrombus formation [21]. ROS production is induced via inflammation via CD36 and PCSK9 binding, which stimulates the MAPK, Src, and NOX2 axis [23]. Furthermore, platelet binding with its agonists (ADP, collagen, and thrombin) raises PCSK9 levels, which in turn increases GPIIb/IIIa and P-selectin, all of which are crucial for adhesion and activation of the platelet [24]. In addition, PCSK9–LOX-1 cross-talk has also been discovered to have a role in mediating atherosclerotic inflammation [25]. In Figure 1, we summarized the roles of PCSK9 during atherosclerosis development that lead to cardiovascular events from initiation, progression, and aggravation, which involves its effect on lipid metabolism, inflammation, and platelet activation.

## 3. In Vitro and In Vivo Model of Study on PCSK9 and Platelet Activation

### 3.1. In Vitro Studies Using Isolated Platelets Model

Beyond its lipid-lowering effect, accumulative evidence has shown the implication of PCSK9 in preventing and slowing down atherosclerosis disease progression by being involved in platelet activation [20]. Hence, several in vitro studies have been done to explore the potential roles of PCSK9 in platelet activation-induced cardiovascular disease, whereby almost purified human platelets were used as a model of study [21,23,26]. For example, Petersen-Uribe et al.’s study had showed how PCSK9 was expressed and released by platelets and promotes atherothrombosis and the inflammation process during atherosclerosis progression [26]. Human platelets were used for their in vitro model of study. In short, venous blood from donors was collected in an acid citrate dextrose (ACD) buffer [26]. Platelet-rich plasma (PRP) was obtained after centrifugation [26]. The PRP was centrifuged once more, and the supernatant was discarded [26]. The pellet was then revived in Tyrode’s solution (pH 7.4, containing 1 mM CaCl2) to obtain a high-purity isolated platelet, and the platelet count was measured using a hematology analyzer [26]. Further, activated platelets models were obtained by stimulating isolated platelets with a platelet agonist, i.e., adenine diphosphate (ADP) and collagen [26]. Next, monocytes that were extracted from mouse spleen were co-cultured with human-activated platelets to form macrophages and foam cells [26]. The human recombinant of PCSK9 and anti-PCSK9 were then used to evaluate the effect on thrombus formation, platelet aggregation, monocyte differentiation into foam cells, and monocyte migration, by which all led to the development of atherosclerosis progression-induced coronary artery disease [26]. In addition, cultivated human liver cells (HepG2) were also used as model of study to compare the expression of PCSK9 expression by immunoblotting assay [26].

Next, in an effort to determine a plausible mechanism underpinning PCSK9 and platelet activation during atherosclerosis progression, Cammisotto et al. also used the purified human platelets [23]. The isolation process followed the same steps as the work by Petersen-Uribe et al. In addition, they added the prostaglandin E1 (PGE1, 1 µM) to prevent platelet activation. Platelet pellets were then sonicated three times in RIPA lysis buffer containing a phosphatase and protease inhibitors [23]. Thereafter, the samples were subjected for co-immunoprecipitation or phosphorylation assay by SDS-PAGE Western blotting [23]. An ex vivo platelet aggregation study was done to induce platelet activation with collagen and subsequent in vitro platelet recruitment was done to evaluate thrombus formation [23]. Results showed that high levels of PCSK9 in the circulation are linked to enhanced platelet activation and thrombus formation, which involves the molecular activation of CD36 and NOX2 [23]. They also discovered that PCSK9 interacts with CD36 using a co-immunoprecipitation analysis, implying that PCSK9 activates platelets by directly binding to CD36 platelets and initiating the subsequent cascade, including ROS generation by activated NOX2, to cause platelet aggregation [23].

In agreement with Cammisotto et al.’s finding, Qi et al. discovered that the PCSK9 and CD36 receptor have a direct relationship that enhances platelet activation resulting in the activation of essential signaling cascades for platelet aggregation [21,23]. PCSK9 directly increases platelet activity and thrombosis by interacting with the CD36 receptor on platelets and causing activation of downstream signaling cascades [21]. However, PCSK9 alone cannot induce platelet aggregation, even at a concentration of 10 g/mL, but concentrations less than 10 g/mL, i.e., 0.3–0.8 g/mL that indicate normal PCSK9 plasma concentrations, have been discovered to increase platelet aggregation after induction by agonist compounds, namely, ADP, thrombin, or collagen [27,28].

In Qi et al.’s model of study, they were using both humans and mouse platelets [21]. The human platelet-rich plasma (PRP) was collected by drawing blood from the antecubital vein and mixing it with acid citrate dextrose buffer [21]. Column sepharose 2B was then used to filter PRP and Tyrode’s solution (pH 7.35) was used to homogenize and extract platelets [21]. Alternatively, platelets can also be collected and reconstituted in Tyrode’s solution after centrifugation at 900 g for 10 min [21]. For mouse platelets, mice were anesthetized, and then using a 21-gauge needle, 180 μL of blood was taken through the inferior vena cava into 20 μL of acid citrate dextrose solution [21]. PRP was then collected by centrifugation of the whole blood at 80 g for 10 min [21]. The PRP that has been successfully collected was suspended into 2 volumes of Tyrode’s solution [21]. A co-immunoprecipitation and phosphorylation activity assay by Western blotting of human and mouse platelets then successfully revealed the specific binding of PCSK9 and CD36, which stimulates Src, ERK5, and JNK, enhancing ROS production and promoting the activation of the p38/cPLA2/COX-1/TXA2 signaling cascades [21].

### 3.2. In Vivo Studies Using Animal Models

Animal models of cardiovascular disease have been developed to conduct a comprehensive study of PCSK9′s pleiotropic involvement in cardiovascular disease progression, particularly in platelet activation. Several types of in vivo studies have employed genetic modification followed by the disease induction model. To date, the animal models that have been used to study the molecular mechanistic functions of PCSK9 and platelet activation are mice and rabbits. In this section, we summarize some of the in vivo studies on PCSK9 and platelet activation.

#### 3.2.1. Mouse

Mice are the most frequently used animal model for human disease research [29]. Mice and humans are biologically highly similar and share many of the same diseases for genetic reasons [29]. Mice can be genetically modified to imitate almost any disease or condition in humans [29]. The finding of PCSK9’s key role in lipid metabolism was made possible by studies conducted on gene-modified mice [12]. For example, adenovirus-induced PCSK9 overexpression in a mouse model leads to lower hepatic LDLR expression and hypercholesterolemia, while ablation of the PCSK9 gene results in increased hepatic LDLR expression and lower circulating LDL-c levels. [30]. Thereafter, Camera et al. found that PCSK9-/- mice had less FeCl3 injury-induced carotid artery thrombosis with unstable non-occlusive thrombus development, implying reduced platelet activation [31]. Platelet activation is initiated by arterial damage in these mice, as evidenced by enhanced P-selectin expression, glycoprotein (GP) IIb/IIIa activation, and platelet–leukocyte aggregation [31].

In line with Camera et al., Dwivedi et al.’s study has demonstrated that the sepsis-induced hypercoagulation state is worsened in PCSK9-overexpressing mice, as shown by increasing the thrombin–antithrombin complex and decreasing plasma protein C levels, suggesting that upregulation of PCSK9 is positively correlated with blood coagulation [32]. Following that, Wang et al. found that after inferior vena cava ligation, PCSK9-/- mice generated substantially smaller venous thrombus than wild-type mice [33]. Next, the recent in vitro results studied by Qi et al. have revealed that the underlying reason for the increase in platelet aggregation activity occurs through interaction between PCSK9 and CD36 receptor that results in promoting downstream signaling pathway activation, namely, ERK5, Src, and JNK, to increase ROS production and p38/cPLA2/COX-1/TXA2 activation [21]. Because PCSK9 degrades when bound to LDLR, they employed LDLR-/- mice to enhance PCSK9 plasma levels [21]. They compared LDLR-/- and wild-type (WT) mice’s plasma PCSK9 and lipid levels and discovered that LDLR-/- mice had significantly higher plasma PCSK9 [21]. PRP from LDLR-/- mice demonstrated greater platelet aggregation caused by ADP compared to WT animals; however, the enhancing impact was decreased by evolocumab, a PCSK9 monoclonal antibody [21]. To confirm that PCSK9 promotes thrombosis in vivo, using intravital microscopy, they evaluated FeCl3-injured thrombus development in mesenteric arterioles in LDLR-/- and WT mice with/without evolocumab or PCSK9 [21]. As a result, compared to saline-injected mice, WT mice under the treatment of PCSK9 had more thrombus development in mesenteric arterioles [21]. Furthermore, when compared to WT mice, LDLR-/- mice had more thrombus formation that was reduced by evolocumab [21].

After exploring the molecular mechanism, they tried to investigate a possible medical intervention on PCSK9-inducd platelet activation using a COX-1 inhibitor, aspirin [21]. To test if aspirin could protect mice from PCSK9-enhanced in vivo thrombosis, WT mice were treated with aspirin for 3 days before receiving PCSK9 or saline injections and subsequent FeCl3 injury [21]. According to these findings, aspirin inhibits PCSK9′s enhancing effects on platelet activation and in vivo thrombosis [21]. By developing an animal model of MI, they also investigated whether aspirin could protect mice against PCSK9-enhanced ischemia that was carried on by microthrombosis and MI expansion [21]. The animal model of MI has an important role in the prevention, diagnosis, and treatment of MI in humans [34]. In coronary artery disease research, MI induction can be carried out by several methods, namely, chemical induction, coronary artery ligation, closed-chest method, coronary artery embolization, balloon catheter, ameroid contraction, and cryoinjury-induced MI [34]. Among them, the left anterior descending (LAD) coronary artery ligation is very commonly used in research related to acute MI using mice model [35].

A MI model was established in their study by inducing transmural ischemia in the left anterior descending coronary artery. ST-segment elevation on electrocardiography in groups of mice confirmed the MI mouse model [21]. While PCSK9 can exacerbate MI progression in various ways, the authors employed a platelet deprivation model to ensure that CD36 was deficient in platelets rather than other cells [21]. The left anterior descending coronary artery ligation-induced MI operation was then carried out [21]. PCSK9-pretreated platelets from WT mice greatly increased the infarct region in platelet-depleted WT mice when compared to untreated platelets, while PCSK9-pretreated platelets from CD36-/- mice greatly decreased the infarct region in platelet-depleted WT mice when compared to mice receiving PCSK9-pretreated WT platelets [21]. Likewise, histologic results demonstrated that mice getting PCSK9-pretreated platelets from WT mice had more microthrombi than mice receiving untreated platelets, whereas PCSK9′s negative effects were dramatically reduced in mice getting PCSK9-pretreated platelets derived from CD36-/- mice [21]. Finally, the findings of Qi et al. show that activation of CD36 platelets via PCSK9 exacerbates microvascular blockage and promotes MI [21].

#### 3.2.2. Rabbit

While mice models have been commonly used for PCSK9-induced cardiovascular disease in vivo studies, they have limitations because they lack the central features of human cardiovascular pathophysiology, clinical manifestations, and some intravascular procedures for imaging [36]. As a result, more ideal disease animal models that are closer to human in size and cardiovascular pathophysiology are required, such as the rabbit. Rabbits are hypersensitive to dietary cholesterol, developing severe hypercholesterolemia and aortic atherosclerosis promptly, making the rabbit an ideal model to study atherosclerosis-related disease that is initiated by a hypercholesterolemia condition [37]. Thus, this model will be beneficial to study PCSK9-induced platelet activation during atherosclerosis development. As a result, cholesterol-fed rabbits are commonly utilized in atherosclerosis research than murine or rodent models [37]. The rabbit was actually the first animal used to explore lipoprotein dynamics and atherosclerosis [37]. Anitschkow, a Russian experimental pathologist, reported in 1913 that feeding cholesterol-soluble sunflower oil to rabbits raised blood cholesterol levels and caused atherosclerosis in the arteries within weeks [37]. Laboratory rabbits, especially New Zealand white (NZW) and Japanese white rabbit (JW), have lower plasma total cholesterol (TC) (30–90 mg/dL) at 3–4 months of age than humans because they are herbivores. However, when the rabbits are fed a cholesterol-rich diet, hypercholesterolemia quickly develops (hyperlipidemic rabbits) [37]. Aortic lesions can occur in hyperlipidemic rabbits as soon as 4 to 6 weeks following dietary cholesterol administration [37]. The age of rabbits should be considered because young rabbits are more susceptible to aortic atherosclerosis than old rabbits, although there is no difference in plasma total cholesterol levels [38].

El-Seweidy et al. conducted one study that successfully used the hyperlipidemic rabbit model to investigate PCSK9 and platelet activation [39]. They discovered that a novel natural-derived cholesteryl ester transfer protein (CETP) inhibitor, 10-dehydrogingerdione, suppressed PCSK9 expression and functions in hyperlipidemic rabbits, which was accompanied by a reduction in platelet activation, cellular adhesion inflammatory molecules, and endothelial dysfunction biomarkers when compared to atorvastatin treatment [39]. For this study, 40 mature albino male rabbits (12 weeks old and weighing 1500–2000 g) were employed and kept in a clean and regulated environment [39]. Prior to the experiment, rabbits were given regular water ad libitum and pellets for 2 weeks [39]. Next, after feeding standard rabbit pellets enriched with 0.5% *w*/*w* cholesterol for 90 days, the hyperlipidemia condition was established [39]. Blood was then taken from ear veins on the periphery; triglycerides (TG), total cholesterol (TC), CETP level and activity, high-density lipoprotein cholesterol HDL-c levels, soluble P-selectin (sP-selectin), interferon-gamma (IFN-γ), soluble CD40 ligand (sCD40L), and PCSK9 levels were all measured in the serum [39]. Finally, they reported for the first time that 10-dehydrogingerdione, a naturally derived CETP inhibitor, has a PCSK9-reducing effect, which could explain its cardiovascular preventive roles against atherogenic and coronary risk indexes, as demonstrated by the reduction in atherosclerotic inflammatory and platelet activation markers [39].

In line with this, they conducted a study concentrating on platelet activation biomarker levels as markers (CD40 ligand and P-selectin) in the serum of dyslipidemic rabbits treated with a combination of 10-dehydrogingerdione and policosanol. Both 10-dehydrogingerdione and policosanol are natural compounds that have been shown to inhibit CETP and reduce atherosclerosis [40]. Their research found that the daily administration of 10-dehydrogingerdione and/or policosanol at a dose of 10 mg/kg bw inhibited CETP, increased HDL-C, decreased PCSK9, and decreased platelet activation and inflammation markers such sP-selectin, sCD40L, and interferon-gamma (IFN-γ). Finally, they demonstrated that their combination therapy had a synergistic impact as potential medications in the prevention of atherothrombosis [40].

## 4. Other Potential Model for PCSK9 and Platelet Activation Research

While in vitro human or mouse platelet cultures combined with in vivo studies in mice or rabbits have been recently used for PCSK9 and platelet activation research, there have been various studies on PCSK9-related cardiovascular disease studies employing another in vitro and in vivo model. In vitro, primary cells from mouse aortic endothelial cells (MAEC) and mouse cardiac endothelial cell (MCEC) lines have been successfully used for the investigation of PCSK9 in atherosclerosis development. In vivo, the direct relationship between PCSK9 and atherosclerosis was investigated by utilizing Apolipoprotein E (ApoE) deficient mice. Moreover, a study using an acute myocardial infarction (AMI) rat model was the first to show a relationship between MI and PCSK9. In rabbits, many models have been established to study atherosclerosis-related diseases without using high-fat induction. In cardiovascular disease research, the porcine atherosclerosis model has also been used to examine the molecular activities of PCSK9 as an anti-hypercholesterolemia medication. NHPs were also used to look at the clinical efficacy and potential side effects of a PCSK9 therapeutic therapy. However, those studies still do not include PCSK9 and platelet activation investigations. The experimental and clinical aspects of the physiological features related to platelet activation are not yet covered in this research, since the role of PCSK9 and platelet activation is still relatively new. As a result, current PCSK9 research in cell lines, mice, rats, porcine, and NHPs has focused only on lipid metabolism markers, with no mention of the platelet activation marker. Nevertheless, the subject is interesting to explore, since it may pave the way for future PCSK9 and platelet activation studies.

### 4.1. Mouse Aortic Endothelial Cells (MAEC) and Mouse Cardiac Endothelial Cell (MCEC) Lines

Sun et al. have demonstrated the involvement of PCSK9 in the regulation of lipid metabolism and atherogenesis by utilizing MAEC and MCEC lines to confirm the LDL effects on the atherosclerosis-related genes expression [41]. The MAEC was prepared by isolating mouse primary aortic ECs from C57BL/6J mice and LDb mice [41]. Briefly, primary ECs were collected by dissecting the aorta from the aortic arch to the abdominal aorta and then were separated using 5 mL of 20% PBS-DMEM containing 1000 U/mL of heparin [41]. The ECs were suspended with 2 mL of 20% FBS-DMEM and cultured in a 35 mm collagen type I-coated dish [41]. Cells were plated onto 12-well 0.2% gelatin coated plates in DMEM endothelial cell growth medium containing 20% FBS, 2 mM glutamine, 100 U/mL penicillin, 100 g/mL streptomycin, 1 mM sodium pyruvate, 20 mM HEPES, 1% non-essential amino acids, 50 mM 2-mercaptoethanol, 12 U/mL heparin, and 150 g/mL endothelium cell growth supplement. After 24 h, the media was transferred to serum-free DMEM containing PBS, 20 g/mL LDb-LDL (containing PCSK-9) or 20 g/mL LTp-LDL (without PCSK-9) and incubated for another 24 h [41]. Then, the cells were collected for RNA extraction. In the case of the mouse cardiac EC (MCEC) line, they were purchased from Cedarlane/CELLutions Biosystems (Burlington, NC) [41]. The MCEC line was established using microvascular neonate MCECs that had undergone lentiviral immortalization using human telomerase and the SV40T antigen [41]. The cells were identified as having EC-like characteristics, such as the ability to form microtubes in matrigel, stain positively for CD31, VE-cadherin, and von Willebrand factor-associated antigen, and take up DiI-AcLDL [41]. On a 0.2% gelatin-coated plate, the cells were grown in DMEM with penicillin and streptomycin, HEPES (10 mmoL/L), and 10% FBS [41]. The MCEC lines were then cultured, treated similarly to MAEC, and subjected to RNA and protein extraction [41]. Both results from MAEC and MCEC lines showed that cells treated with LDb-LDL (containing PCSK9) induced a greater expression of pro-inflammatory and pro-autophagy genes, which are important for atherogenic properties, indicating the key role of PCSK9 in promoting atherosclerosis [41]. A similar model could be utilized to further evaluate the platelet activation marker gene and protein expressions for future experiments.

### 4.2. Mouse

Advanced atherosclerotic lesions can occur spontaneously in ApoE-deficient mice even when they are provided with a regular diet [42]. For the first time, Denis et al. demonstrated that ApoE-deficient mice are protected against atherosclerosis by the absence of PCSK9, and PCSK9 overexpression leads to more severe atherosclerotic phenotypes [42]. In their study, they utilized a transgenic mouse with an ApoE-deficient background that expressed no PCSK9, a basal level of PCSK9, or a high level of murine PCSK9 [42]. After six months fed by a regular diet, total plasma lipids, lipid profile, and atherosclerotic lesion size and composition were then evaluated [42]. When compared to WT mice that were fed on a regular diet, ApoE-knockout (KO) mice showed a higher accumulation of total plasma lipids [42]. However, total plasma lipid levels were not significantly impacted by PCSK9 deletion or overexpression, probably due to the potent effects of ApoE deficiency [42]. Interestingly, the aortic cholesteryl esters content was reduced by 39% in ApoE KO with no PCSK9 mice and increased by 137% in ApoE KO with high PCSK9 mice [42]. Furthermore, compared to ApoE KO mice with no PCSK9 or ApoE KO mice with a basal level of PCSK9, ApoE KO with high PCKS9 mice exhibited a highly significant increase in the average plaque size and number [42]. Thus, Denis et al.’s study highlighted a direct relationship between PCSK9 and the development of atherosclerosis using mice with an an ApoE-deficient background [42]. The same established system may be used in future investigations into PCSK9 and platelet activation during atherosclerosis development.

### 4.3. Rat

Some studies have used rats in their research when studying the roles of PCSK9 in cardiovascular disease progression. For the first time, Zhang et al. showed that plasma PCSK9 levels were significantly raised 12 h after AMI, which was subsequently verified by increased liver PCSK9 mRNA levels [43]. The LAD coronary artery was ligated under ether anesthesia to create myocardial infarction rat models [43]. Electrocardiography was used to show ST elevation and hence verify surgical completion [43]. Palee et al. used a similar rat model to study the cardioprotective effects of PCSK9 inhibitors in the rodent acute cardiac ischemia/reperfusion (I/R) model [44]. In brief, rats were euthanized and mechanically ventilated using room air following a tracheotomy along the ventral midline [44]. Throughout the experiment, an electrocardiogram (ECG) was recorded [44]. After a left side thoracotomy, the LAD was ligated with a surgical stitch 2 mm proximal to the origin [44]. Myocardial blanching and an ECG with ST elevation revealed myocardial ischemia [44]. Their research found that providing the PCSK9 inhibitor before ischemia protects the heart from the injuries incurred by I/R [44]. They discovered that cardioprotective processes included increased connexin43 phosphorylation, enhanced cardiac mitochondrial activity, and a reduction in apoptosis, which resulted in reduced cardiac arrhythmia and infarct area, and eventually improved left ventricular performance [44]. These data point to the possibility of using the PCSK9 inhibitor for purposes other than hyperlipidemia [44]. In both of these rat MI models, more work needs to be done to determine the effects of PCSK9 on platelet activation, such as by creating a myocardial injury system in an existing MI rat model and then evaluating the platelet activities.

### 4.4. Rabbit

Instead of high-cholesterol-diet-induced hyperlipidemic rabbits, there is another model that might also be used for PCSK9 and platelet activation research, that is, spontaneous hyperlipidemic rabbits (Watanabe heritable hyperlipidemic (WHHL) [37]. Dr. Watanabe developed the rabbit at Kobe University in Japan in the 1980s, and it is frequently used as a model for human familial hypercholesterolemia (FH) [37]. PCSK9 is high in individuals with FH, which is an autosomal dominant genetic condition associated with increased plasma LDL levels due to LDLR deficiency; moreover, previous research has found that PCSK9 is elevated in people with FH in humans [10]. Because of WHHL, the rabbits are genetically defective in LDLR function; they can develop hyperlipidemia even on a regular standard diet [37]. Serum lipoproteins were characterized by electrophoresis, showing a broad pattern of lipoprotein elevation and decreased lipoprotein in rabbit WHHL [37]. In WHHL rabbits, LDLR dysfunction causes a loss of LDL absorption by the liver and, as a result, an increase in plasma LDL levels, comparable to human FH [37,39]. In addition, WHHL rabbits exhibit metabolic disorders such as insulin resistance and visceral fat deposition [37]. Some WHHL rabbits have been also linked to coronary artery disease and myocardial infarction [37]. Therefore, this WWHL rabbit might be an ideal animal model for studying PCSK9 and platelet activation in the context of atherosclerotic disease progression. However, no study utilizes this animal model yet for investigating PCSK9 functions.

Another rabbit model that has been generated but not used for PCSK9 and platelet activation studies is PCSK9 point mutation rabbits using clustered regularly interspaced short palindromic repeats (CRISPR)/Cas9 technology, which Yan et al. has successfully established [45]. In their research, BLAST was used to examine the functional areas of the PCSK9 protein in humans and rabbits based on PubMed gene protein data [45]. Because the human PCSK9 gene’s 386S (Ser) amino acid essential region was identical to the rabbit PCSK9 gene’s 485S, Yan et al. targeted those certain sequences with single-stranded donor oligonucleotide, synthesized short guide RNAs, and Cas9 mRNA [45]. The embryos were implanted into pregnant rabbits after the gRNA, cas9, and DNA donor were injected into the cytoplasm of rabbit fertilized eggs [45]. On the F0 rabbits, PCR, TA cloning, and off-target detection were used to see if the PCSK9S386A mutation was successful. From 15 F0 rabbits, the sequencing revealed that two of them were PCSK9S386A point mutation heterozygotes and one of them was a PCSK9S386A point mutation homozygote, indicating that the mutation may be inherited in a stable manner [45]. As a result of the CRISPR/Cas9 technique, they were able to successfully establish a rabbit model of PCSK9S386A point mutation, which serves as an animal model for investigating the molecular mechanisms of impaired PCSK9 function and developing effective and reliable diagnosis and treatment measures [45].

### 4.5. Porcine

Advances in genetic engineering have the possibility to build such models in tiny pig strains that can develop hypercholesterolemia under circumstances nearly identical to human hypercholesterolemia that might be linked to PCSK9 study. Furthermore, it has been observed that pigs can develop spontaneous atherosclerosis, which has a pathogenesis comparable to that of humans [36]. For example, the Ossabaw mutant pig animal model can be used to investigate the effect of PCSK9 on atherosclerosis. Ossabaw pigs are rare small pigs with a hereditary vulnerability to metabolic syndrome and coronary atherosclerosis when fed a high-fat diet for lengthy periods of time. Even without a high-fat diet, Ossabaw pigs have the greatest average lipid levels in the mammalian class and are innately prone to vascular disease. Familial hypercholesterolemia and coronary artery plaques grow faster in transgenic Ossabaw mutant pigs, obtained by the transposition of chimpanzee’s DNA, expressing D374Y gain-of-function (GOF) PCSK9 genes [36,46].

The Ossabaw-PCSK9 pig animal model after 6 months of induction can be used for an animal model that perfectly mimics the human condition; this animal can be used to monitor the development of atherosclerosis using magnetic resonance (MR) imaging, computed tomography, optical coherence tomography, and intravascular ultrasound to analyze and evaluate the pathological of atherosclerosis in coronary arteries. Compared to other strains of pigs, PCSK9-GOF pigs have elevated cholesterol, triglycerides, and blood pressure levels at 3 and 6 months. LDL, total cholesterol, and triglyceride levels in Ossabaw-PCSK9 pigs show a significant increase compared to the control group [46]. There was an increase in LDL as much as 250.1 ± 17.4 compared to the control group, which was only 36.3 ± 21.0. Peripheral arteries in the PCSK9-GOF group showed medial thickening of 2.0 vessels/mm compared to 1.5 vessels/mm in the control group. Plaque formation in PCSK9-GOF was 20% higher than control, at 10% at 9 months of age [47]. The model is genetically a novel tool for testing therapeutic interventions in the context of the development and treatment of advanced coronary artery disease, including study about PCSK9 and platelet activation [46,47].

Another rabbit model that has been used to research about PCSK9-induced atherosclerosis is a Yucatan minipig, which can express D374Y-PCSK9. Decreased hepatic LDLR levels and LDL clearance, hypercholesterolemia, and spontaneous development of atherosclerotic lesions were observed in D374Y-PCSK9 transgenic pigs by noninvasive imaging. In a transgenic Yucatan minipig with multiple insertions of the transgene, the mechanism by which the Yucatan minipig D374Y-PCSK9 transgene produces hypercholesterolemia was evaluated. The liver LDLR level was reduced by 90% compared to the control group; moreover, this transgenic model has lower HDL levels compared to other pig strains. The thickening of the aorta reaches 80%, and it can be used for further studies of atherosclerosis [36]. Therefore, the Yucatan minipig pig as an animal model of atherosclerosis with D374Y-PCSK9 expression can potentially be used for further research in PCSK9 and platelet activation during atherosclerosis disease progression.

### 4.6. Non-Human Primate

Non-human primates (NHPs) were used in research to assess the clinical efficacy and possible side effects of potential PCSK9-modifying drugs, bringing us closer to the clinical use of PCSK9-targeted therapy in humans. A study by Kamenetsky et al. involved not only mice and rats but also NHPs as their model of study to evaluate the inhibition effect of PCSK9 via an siRNA mechanism [48]. They developed and produced multiple siRNA therapeutic agents to downregulate PCSK9 mRNA in rats, mouse, and NHPs [48]. To achieve efficient hepatocyte distribution in vivo, these siRNAs were delivered utilizing a lipidoid nanoparticle (LNP) [48]. They were able to investigate the effects of PCSK9 downregulation on PCSK9 mRNA, plasma PCSK9 protein, hepatic LDLR protein, total blood cholesterol, HDL-c, and LDL-c concentrations in various species using this method. Briefly, they showed that a single 30-min infusion of two distinct formulation PCSK9 siRNAs led in a highly significant, rapid, precise, and durable reduction of plasma apoB, LDL-c, and PCSK9 protein levels, excluding HDL-c or TGs, in trials with cynomolgus monkeys. LDL-c was reduced by 50–60% within 48 h after siRNA-mediated reduction of PCSK9 protein and mRNA after administration, and this reduction persisted for nearly 3 weeks [48]. These in vivo investigations show that siRNA-mediated PCSK9 inhibition reduces plasma LDL-c but not HDL-c in NHPs. Their findings suggest PCSK9 as a therapeutic target for siRNA intervention and offer a treatment strategy for lipid-lowering therapy that can be used to treat cardiovascular disease patients with hypercholesterolemia.

NHPs were also reported to be an animal model for evaluating the genome editing-based technology, CRISPR/Cas9, in silencing the PCSK9 gene permanently. The principle of gene therapy in silencing PCSK9 utilizing CRISPR/Cas9 tools has been well-reviewed elsewhere [14]. Wang et al. used this method in NHPs for the first time in vivo [49,50]. They disclosed the use of an adeno-associated virus (AAV) containing the genetic coding for a meganuclease that targets genetic changes, resulting in a reduction in PCSK9 protein [49,50]. The researchers tested their approach on rhesus macaques, finding dose-dependent decreases in PCSK9 levels of up to 84% and LDL-c reductions of up to 60% [49,50]. However, there were a vast amount of off-target sites where inadvertent editing occurred. After a thorough investigation of off-target regions, it was discovered that the nuclease might produce reduced (but nonetheless measurable) off-target editing while maintaining LDL-lowering effects [49,50]. While utilizing a meganuclease offers the advantage of allowing adeno-associated viruses to be utilized rather than adenovirus, it also has significant off-target effects and an immune response.

As a result, Musunuru et al. upgraded their research, which is the most advanced PCSK9 in vivo editing attempt to date [51]. The employment of a specific base editor resulted in a very specific one-nucleotide exchange rather than double-strand breaks in the DNA, which is the most significant distinction from earlier studies [14]. Adenine base editing was found to be very effective in shutting down PCSK9 gene expression and function in the liver of a cynomolgus monkey in their trials, with over 60% editing achieved [51]. PCSK9 levels in the blood have been decreased by approximately 90%, surpassing all existing LDL-lowering treatment medicines, according to their data (which vary from chronic once-daily to twice-yearly dosing) [51]. Therefore, they have the potential to be the one-and-done therapy for the long-term treatment of disease. Although the lengthy endurance of CRISPR-based liver editing has yet to be determined, there are no signs of the distortion of liver editing’s pharmacodynamic effects in cynomolgus monkeys in their long-term study [51]. Thus, Musunuru et al. have provided us an excellent study of PCSK9 as lipid-lowering agent utilizing NHPs, which may open the way for future study of PCSK9 as antiplatelet agents.

## 5. Discussion and Conclusions

Developing potential new therapies related to PCSK9 and platelet activation in cardiovascular disorders demands an experimental approach to understand the fundamentals of the disease mechanism. Experimental in vitro models using isolated human platelet and in vivo models using genetic modified animal models, followed with the induction of a disease model, i.e., myocardial infarction, atherosclerosis, and dyslipidemia, were useful for PCSK9 research. Table 1 summarizes the experimental models used to study PCSK9-induced platelet activation in cardiovascular disease research. Comprehensive studies of in vitro and in vivo data are required to deliver the new information that can be useful for PCSK9-related drug development. An ideal research model of cardiovascular disease should represent the human condition metabolically and pathophysiologically. Therefore, studys using an in vitro model should always be further confirmed in an in vivo study that more closely mimics the human condition.

Researchers may now modify particular targets in genes or proteins that have a role in disease, especially with the emergence of genetically engineered animal models. As a result of these circumstances, a wide range of models of prospective targets for enhanced interventions have been discovered. Moreover, studies using animal models do provide new perspectives into essential roles of PCSK9 and cardiovascular disease research activity, particularly in platelet activation context. However, there is still no model that is completely ideal for all studies, considering the chronic nature of vascular disease (myocardial infarction, atherosclerosis, and dyslipidemia). As a result, choosing the right animal model to research various aspects of vascular disease is critical. Otherwise, many intriguing study findings may not be successful when applied to human trials. An agreement on experimental models suitable for various vascular disease studies would be a practical and effective technique for advancing research on PCSK9 and platelet activation.

For example, mice are the most commonly used animal model in human disease models, including for the early investigation of PCSK9 and platelet activation mechanistic study. Usually, instead of mice, rats will be another selected model because both mice and rats are affordable, simple to buy, and easy to maintain [29]. However, because murine and rodent models lack CETP and carry the majority of their plasma cholesterol in HDL, the murine and rodent models could not be used as an ideal research model in the context of PCSK9-induced platelet activation research that naturally requires the pathophysiological features of atherosclerosis disease development [52]. Thus, determining whether PCSK9 downregulation will only reduce LDL-c and not HDL-c will be difficult using murine or rodent models [52]. As a result, research in a more relevant model, such as rabbit, porcine, and NHPs, has been developing. However, the study focusing on PCSK9 and platelet activation using porcine and NHP models is still limited, while actually they are indeed a more robust model to study PCSK9-induced platelet activation in dyslipidemia or atherosclerotic animal disease model. Table 2 summarizes the prospective animal models that can be used for future PCSK9-induced platelet activation in cardiovascular disease research.

Nevertheless, although animal models can resemble the human disease model, there are still some differences in anatomical and physiological features. Because of these differences, the results of studies using animals cannot be representative of results without clinical trials in humans [53]. Therefore, to complete the in vitro and in vivo models, clinical data from humans is also needed for a thorough analysis of PCSK9-induced platelet activation in cardiovascular disease in the future.

## Figures and Tables

**Table 1:** The experimental models used to study PCSK9-induced platelet activation in cardiovascular disease research.

Study	Model (Cell/Animal)	Method	Results
In vitro	Purified human platelets; cultivated human liver cells (HepG2)	Platelet isolation, SDS-PAGE Western blot assay, platelet aggregation assay, thrombus formation assay, monocyte migration, and monocyte differentiation assay	PCSK9 was expressed and released by platelets that promote atherothrombosis and inflammation process during atherosclerosis progression [26].
	Purified human platelets	Platelet isolation, co-immunoprecipitation and phosphorylation assay by SDS-PAGE Western blot assay, platelet aggregation assay, and thrombus formation assay	High levels of PCSK9 in the circulation are linked to enhanced platelet activation and thrombus formation, which involves the molecular activation of CD36 and NOX2 [23].
	Purified human and mice platelets	Platelet isolation, co-immunoprecipitation or phosphorylation assay by SDS-PAGE Western blot assay, and platelet aggregation assay	Specific binding of PCSK9 and CD36, which stimulates Src, ERK5, and JNK, enhancing ROS production and promoting the activation of the p38/cPLA2/COX-1/TXA2 signaling cascades [21].
In vivo	Mouse	Establishment of PCSK9-/- mice, FeCl_3_ injury-induced carotid artery thrombosis;	Platelet activation was impeded when PCSK9 was downregulated [31].
		PCSK9-expressing mice, sepsis-induced hypercoagulation;	Upregulation of PCSK9 is positively correlated with blood coagulation [32].
		PCSK9-/- mice inferior vena cava ligation;	PCSK9-/- mice developed significantly smaller venous thrombus than wild-type mice [33].
		LDLR-/- mice, ischemia-induced microthrombosis and MI expansion by establishing animal model of MI, CD36-/-mice	The activation of CD36 platelets via PCSK9 exacerbates microvascular blockage and promotes MI [21]
	Rabbit	Hyperlipidemic rabbits induced by high-fat diet of 0.5% w/w cholesterol for 90 days;	A novel natural-derived cholesteryl ester transfer protein (CETP) inhibitor, 10-dehydrogingerdione, suppressed PCSK9 expression and functions in hyperlipidemic rabbits, which was accompanied by a reduction in cellular adhesion inflammatory molecules, platelet activation, and endothelial dysfunction markers when compared to atorvastatin treatment [39].
		Hyperlipidemic rabbits induced by high-fat diet of 0.5% w/w cholesterol for 90 days;	Daily administration of policosanol and/or 10-dehydrogingerdione at a dose of 10 mg/kg bw inhibited CETP, increased HDL-C, decreased PCSK9, and decreased platelet activation and inflammation markers such sCD40L, sP-selectin, and interferon-gamma (IFN-γ) [40].

**Table 2:** The prospective animal model that can be used for future PCSK9-induced platelet activation in cardiovascular disease research.

Cells/Animals	Model of Study	Results
Cell-based	Mouse aortic endothelial cells (MAEC) and mouse cardiac endothelial cell (MCEC) line;	Cells treated with LDb-LDL (containing PCSK9) induced greater expression of pro-inflammatory and pro-autophagy genes, which are important for atherogenic properties, indicating the key role of PCSK9 in promoting atherosclerosis [41].
Mouse	Transgenic Apolipoprotein E (ApoE) knockout (KO) mouse;	Direct relationship between PCSK9 and the development of atherosclerosis in an ApoE-deficient mouse background [42].
Rat	Acute myocardial infarction (AMI) model;	Plasma PCSK9 concentration was significantly raised 12 h after AMI, which was subsequently verified by increased liver PCSK9 mRNA levels [43]
	acute cardiac ischemia/reperfusion (I/R) model;	PCSK9 inhibitor treatment before ischemia protects the heart from the injuries incurred by I/R [44].
Rabbit	Spontaneous hyperlipidemic rabbits (Watanabe heritable hyperlipidemic (WHHL);	WHHL rabbits are genetically defective in LDLR function and can develop hyperlipidemia even on a regular standard diet. Some rabbit WHHL has been linked to coronary artery disease and myocardial infarction [37].
	PCSK9 point mutation rabbits utilizing the CRISPR/Cas9 technology;	Established a rabbit model of PCSK9S386A point mutation, which serves as an animal model for investigating the molecular mechanisms of impaired PCSK9 function and developing effective and reliable diagnosis and treatment measures [45].
Porcine	Ossabaw-PCSK9 GOF pig animal model;	PCSK9-GOF pigs saw an increase in cholesterol, triglycerides, and blood pressure levels at 3 and 6 months. LDL, total cholesterol, and triglyceride levels in Ossabaw-PCSK9 pigs showed a significant increase compared to the control group. Peripheral arteries in the PCSK9-GOF group showed medial thickening and plaque formation in PCSK9-GOF was higher than control [36,46].
	D374Y-PCSK9 Yucatan transgenic pigs;	Yucatan minipig D374Y-PCSK9 transgene produces hypercholesterolemia. The liver LDLR level was reduced by 90% compared to the control group; moreover, this transgenic model has lower HDL levels compared to other pig strains. Thickening of the aorta reaches 80% so that it can be used for further studies of atherosclerosis [36].
Non-human primate	Cynomolgus macaques;	siRNA-mediated PCSK9 inhibition reduces plasma LDL-c but not HDL-c in NHPs. [48].
	Rhesus macaques;	Adeno-associated virus carrying the genetic code for a meganuclease targeting genetic alterations, thus resulting in PCSK9 protein reduction. The researchers found dose-dependent reductions in PCSK9 levels of up to 84%, as well as LDL-c reductions of up to 60%. [49,50].
	Cynomolgus macaques;	Knocking down PCSK9′s gene expression and function in the liver of the cynomolgus monkey, with over 60% editing achieved. According to their findings, PCSK9 levels in the blood have been reduced by nearly 90% [51].

## Data Availability

The authors will provide the references used for the literature study upon request.
